# Balancing emotional processing with ongoing cognitive activity: the effects of task modality on intrusions and rumination

**DOI:** 10.3389/fpsyg.2015.01275

**Published:** 2015-08-27

**Authors:** Antonietta Curci, Emanuela Soleti, Tiziana Lanciano, Valentina Doria, Bernard Rimé

**Affiliations:** ^1^Department of Education, Psychology, Communication, University of Bari Aldo Moro, Bari, Italy; ^2^Institute of Psychological Sciences, University of Louvain, Louvain-la-Neuve, Belgium

**Keywords:** intrusion, rumination, working memory, verbal vs. visuospatial mode, emotional processing

## Abstract

In the present paper we aimed to show that competition for resources between post-emotional processes and the execution of a cognitive task will result in two possible effects: (1) an impairment of the cognitive task in the short run and (2) an elongation of intrusions and rumination in the long run. The outcome of this competition is influenced by the interaction of the modality (verbal vs. visuospatial) of cognitive tasks run in the aftermath of an emotional experience and the nature (verbal vs. visuospatial) of the same experience. Non-clinical participants were given a working memory task (OSPAN vs. an analog Visual task) before and after the presentation of negative vs. neutral material (a novel excerpt in Experiment 1 and a video clip in Experiment 2). Intrusions and rumination were measured after a 24-h delay. Rumination was also assessed immediately after the experimental induction. Results showed that exposure to verbal negative material impaired verbal performance (Experiment 1); by contrast, exposure to visual negative material impaired both verbal and visuospatial performance (Experiment 2). Intrusions were only affected by the emotional valence of the original experience, while performing a visuospatial task resulted in enhanced rumination only after exposure to verbal emotional material. The findings of both experiments suggest that emotional processing spreads over time in balance with ongoing cognitive activities, and, in such a balance, the visuospatial processing mode tends to prevail over verbal engagements.

## Introduction

In present study we focused on the cognitive remainders of emotional experiences, which consist of intrusive images and ruminative thoughts (Watkins, 2008). We aimed to investigate the impact of the individual’s availability of cognitive resources upon intrusions and rumination. Following studies on traumas and post-traumatic stress disorder (PTSD), intrusions are conceptualized as visual images and sensory impressions which seem to be happening *here and now*, rumination is considered as a class of evaluative thoughts concerning a traumatic experience (Hackmann et al., 2004).

As for traumas, after an upsetting event individuals engage in memory processing that takes the form of intrusions and rumination, and which is much more demanding as the subjective level of distress induced by the experience increases (Kron et al., 2010). Both intrusions and rumination are associated with a struggling effort to keep disturbing material out of one’s consciousness and the processes require that cognitive resources are exploited until a resting state is attained (Cann et al., 2011; Lindstrom et al., 2011; Curci and Rimé, 2012).

In recent years, both correlational and experimental research work has shown that intrusions and rumination are affected differently by the individual’s availability of cognitive resources. Recent studies have proposed that “cold” executive functions such as working memory (WM) are implicated in “hot” processes of emotion regulation (for a review, see Hofmann et al., 2012). More specifically, high WM capacity has been found to be related with the successful regulation of intrusions and unwanted thoughts concerning emotional contents (Klein and Boals, 2001; Brewin and Beaton, 2002; Brewin and Smart, 2005; Curci et al., 2013). Evidence on this regard converge on explicative models connecting the individual’s availability of cognitive resources with the development of intrusions and rumination concerning an emotional event.

### Theoretical Approaches to Intrusions

In this section we will present theoretical accounts on intrusions of traumatic material and their link with the cognitive system, and will then go on to discuss ruminative thoughts in the subsequent section.

The so-called “modality” hypothesis of the development of intrusions (Hagenaars et al., 2010; Brewin and Burgess, 2014) postulates that when individuals experience traumatic events a predominance of a sensory/visuospatial encoding increases the frequency of later intrusions as compared with a verbal encoding (Holmes et al., 2004; Holmes and Bourne, 2008; Krans et al., 2010). Following this account, Holmes et al. (2009) found that playing a visuospatial task, i.e., the computer game “Tetris,” after the presentation of traumatic material prevents memory consolidation and reduces involuntary flashbacks while the execution of a verbal task, i.e., the game “Pub Quiz” has the opposite effect of increasing unwanted intrusions (Holmes et al., 2010).

By contrast, the “distraction” hypothesis (Gunter and Bodner, 2008; Krans et al., 2009) proposes that intrusions, like any other autobiographical formation, are the result of a constructive process that attempts to integrate incoming information into the autobiographical knowledge base (Conway and Pleydell-Pearce, 2000; Conway et al., 2004). The process determines the allocation of attentional resources through concurrent tasks. It follows that any task diverting attention from the active elaboration of an emotional experience can reduce the amount of intrusions related with this experience. Confirmation of this account has been provided in studies that have found a decrease in intrusion frequency as a result of both verbal and visuospatial interference (e.g., Krans et al., 2009).

The two models outlined above propose a different consideration for WM processes implied in the persistence of intrusions. While the modality hypothesis assigns a central role to visuospatial processing in the encoding of traumatic experiences, the model supporting the distraction hypothesis emphasizes the influence of executive control processes in the formation and maintenance of unwanted intrusions.

### Cognitive Processing and Rumination

Rumination is another common consequence of upsetting events (Ehlers and Clark, 2000; Evans et al., 2007) and might persist for a long time after the original experience. Rumination can have different forms (Watkins, 2008), but when it follows emotional experiences it mainly consists of a class of repetitive thoughts contributing to the processing of these experiences (Horowitz, 1986; Tait and Silver, 1989; Harber and Pennebaker, 1992; Janoff-Bulman, 1992).

A link between rumination and WM functioning has been theorized by Baddeley (2013), in his conceptualization concerning depressive phenomena: Rumination associated with depression is considered as a consequence of a malfunctioning of the central executive and the episodic buffer of the WM model. An increasing number of empirical studies have assessed the relationship between the individual’s tendency toward depressive rumination and inhibition deficits (Joorman, 2006; Whitmer and Banich, 2007; Joormann and Gotlib, 2008), biases in refreshing (Bernblum and Mor, 2010), and failure to switch the attentional focus (De Lissnyder et al., 2012). However, these studies were mainly concerned with depressive rumination or rumination associated with emotional disorders, not with rumination following daily emotional experiences.

All the above reviewed studies suggest that an individual’s availability of cognitive resources influences the persistence of both intrusions and rumination. However, the nature and extent of this influence is different for intrusions and rumination. In our previous study (Curci et al., 2013), we showed that, being a form of cognitive elaboration, rumination loads on the individual’s resources more than intrusions, which correspond to sensory impressions and images of little if any conceptual content (Hackmann et al., 2004). In the present study, we maintain that intrusions and rumination are two distinct albeit related phenomena. Intrusions are mostly influenced by the emotional intensity of the original experience regardless of the visuospatial vs. verbal nature of this experience. Rumination is mostly affected by the cognitive elaboration of declarative contents and associated autobiographical context. As such, rumination entails a verbal processing that might interfere with the concomitant execution of other verbal tasks.

### Toward an Integrative Perspective

In the present paper we propose a new model attempting to reconcile and integrate the above outlined perspectives concerning the development of intrusions (the modality and distraction hypotheses) and we aim to extend this integrative perspective to rumination. Our idea is that the formation and maintenance of intrusions and rumination is an effect of the distribution of cognitive resources, and, following the distraction hypothesis, we suggest that the allocation of resources is determined by a central executive mechanism (Baddeley and Hitch, 1974; Baddeley, 1986). This mechanism has been found to be responsible for a modality-specific interference between a WM task and distress symptoms and their regulation (Andrade et al., 1997; Baddeley and Andrade, 2000; Kemps and Tiggemann, 2007). Instead, we suggest that such an interference intervenes during the processing of the original experience and has both immediate (i.e., failures in competing tasks) and/or delayed effects (i.e., persistence of intrusions and rumination).

We believe that the central mechanism checks the congruence/incongruence between the nature of the emotional material to which the individual is exposed (verbal vs. visuospatial) and the modality of a concomitant cognitive task (verbal vs. visuospatial), so that a higher competition is expected in the case of congruence between emotional material and task modality. However, when conflict arises, the same mechanism allocates the needed resources *during the course of time*. To illustrate, following a verbal emotional experience the individual’s cognitive resources will be depleted when a verbal task is concurrently run. In contrast, cognitive elaboration of visuospatial emotional material will overload the system when it is simultaneously engaged in a visuospatial task. The central mechanism will overcome the restriction of resources by spreading the process in the long run. Therefore in an attempt to obtain optimal performance in a concomitant task, individuals might leave the emotional experience unprocessed (or partly processed), so that they will be repeatedly confronted with emotional remainders over time (Curci et al., 2013).

Finally, and in line with the modality hypothesis, we acknowledge that in a competition for cognitive resources, processing a visual emotional experience will tend to prevail over any cognitive engagement in the long run. Hence, we maintain that visual emotional material will sustain intrusions and rumination in the long run, regardless of the sensory modality of the competing WM task. The same will not hold for the verbal contents of emotional experiences. The conflict between a concomitant verbal task will protract rumination, as a result of interference in verbal processing, but will not necessarily result in a significant prolongation of intrusions over time.

### Overview and Hypotheses

In two experiments here described, we investigated the impact of the individual’s availability of cognitive resources upon intrusions and rumination. In conformity with our previous study (Curci et al., 2013), we propose that a competition for resources induced between the post-emotional processes and the execution of a concomitant cognitive task will result in two possible effects. These are (1) an impairment of the cognitive task in the short run and (2) an elongation of intrusions and rumination in the long run. The outcome of this competition will depend on a cognitive balance between the resources allocated in immediate and delayed processes. An important novelty of the present study is that we considered the role of a verbal vs. visuospatial processing modality in that competition of resources. In the first experiment, the emotional material was a novel excerpt; in the second experiment, following the trauma film paradigm (Holmes and Bourne, 2008), we adopted a short emotional clip.

Based on the previously reported reasoning and the reviewed findings, we formulated the following hypotheses:

(1) In a test–retest design, individuals exposed to neutral material will report a practice effect upon WM performance; individuals exposed to emotional material will not improve their performance when the task shares the same modality (verbal vs. visuospatial) of emotional material (Andrade et al., 1997; Baddeley and Andrade, 2000; Kemps and Tiggemann, 2007; Whitmer and Banich, 2007; Joormann and Gotlib, 2008; Bernblum and Mor, 2010; De Lissnyder et al., 2012; Curci et al., 2013).
(2) For individuals exposed to verbal emotional material, rates of long-term rumination will be higher when the concurrent WM task shares the same (verbal) modality of emotional material than when the task has a visuospatial modality; intrusions will be only affected by the emotional valence of the original material (Hackmann et al., 2004; Curci et al., 2013).
(3) For individuals exposed to visual emotional material, intrusions and rumination will persist over time regardless of the WM task modality (verbal vs. visuospatial; Hackmann et al., 2004; Curci et al., 2013).

## Experiment 1

### Materials and Methods

#### Participants and Design

The studies were approved by the Ethical Committee of the Department of Education, Psychology, Communication, University of Bari Aldo Moro. Participants provided written informed consent to participate in the two experiments. A total of 120 participants (54% women; *M*_age_ = 34.70, SD = 11.48) were involved in experiment 1. The study adopted a 2 × 2 between subjects design, with Emotional valence (Negative vs. Neutral) and WM Task modality (Verbal vs. Visuospatial task) as independent variables. The dependent variables of the study are: (1) The index of performance at a WM task, (2) the Impact of Event Scale-Revised (IES-R; Weiss and Marmar, 1996), (3) the Event Related Rumination Inventory (ERRI; Cann et al., 2011). A repeated measure assessment was adopted for WM performance and ERRI indices.

#### Procedure

The procedure was similar to that adopted in the study by Curci et al. (2013). Participants were recruited through a snowball sampling method (Biernacki and Waldorf, 1981): Four undergraduate students were involved in the study as part of their assignment. They were unaware of the research hypotheses and asked to identify other individuals willing to take part in a study on general cognitive abilities. A total 124 participants were invited to take part in the experimental session but four of them abandoned the session before completing it. The distribution of the sample across conditions is reported in the heading row of Table 1. Participants were tested individually and the whole session lasted 50 min on average.

**TABLE 1:** **Chemical parameters of influent solution**.

									***F* (partial **η^2^**)**						
	**Negative *M* (SD)**	**Negative *M* (SD)**	**Neutral *M* (SD)**	**Neutral *M* (SD)**	**Emotional valence**	**Task**	**Test–retest**				
**Measures (min-max; Cronbach’s alpha)**	**Verbal (*n* = 31)**	**Visuospatial (*n* = 31)**	**Verbal (*n* = 31)**	**Visuospatial (*n* = 27)**	**(a)**	**(b)**	**(c)**	**(a × b)**	**(a × c)**	**(b × c)**	**(a × b × c)**
**Screening phase**										
RRS-Brooding (1–4; 0.79)	2.08 (0.45)	2.05 (0.48)	2.10 (0.53)	2.14 (0.52)	0.43 (0.00)	0.00 (0.00)	–	0.18 (0.00)	–	–	–
RRS-Depression (1–4; 0.77)	2.25 (0.69)	1.87 (0.53)	2.08 (0.64)	2.10 (0.53)	0.09 (0.00)	2.74 (0.02)	–	3.32 (0.03)	–	–	–
RRS-Reflection (1–4; 0.71)	1.94 (0.64)	2.32 (0.65)	1.94 (0.66)	2.00 (0.64)	1.82 (0.02)	3.38 (0.03)	–	1.82 (0.02)	–	–	–
PA-T (0–4; 0.77)	2.77 (0.49)	2.88 (0.52)	2.88 (0.51)	2.87 (0.47)	0.28 (0.00)	0.31 (0.00)	–	0.41 (0.00)	–	–	–
NA-T (0–4; 0.87)	1.22 (0.62)	1.37 (0.73)	1.35 (0.76)	1.28 (0.75)	0.03 (0.00)	0.10 (0.00)	–	0.71(0.01)	–	–	–
**Experimental phase**										
PA-S test (0–4; 0.88)	2.73 (0.56)	2.79 (0.75)	2.68 (0.82)	2.90 (0.58)	2.16 (0.02)	1.01 (0.01)	68.72**** (0.37)	0.00 (0.00)	4.01* (0.03)	0.07 (0.00)	0.85 (0.01)
PA-S retest (0–4; 0.91)	1.92 (0.75)	2.08 (0.87)	2.34 (0.96)	2.35 (0.78)							
NA-S test (0–4; 0.90)	0.55 (0.60)	0.46 (0.62)	0.49 (0.72)	0.51 (0.70)	4.01* (0.03)	0.05 (0.00)	0.31 (0.00)	0.36 (0.00)	12.63**** (0.10)	1.03 (0.01)	0.04 (0.00)
NA-S retest (0–4; 0.91)	0.74 (0.84)	0.75 (0.69)	0.24 (0.35)	40 (0.75)							
Emotional intensity (0–10)	4.74 (3.28)	5.23 (3.39)	0.84 (1.46)	1.00 (2.27)	66.18**** (0.36)	0.42 (0.00)	–	0.10 (0.00)	–	–	–
WM performance test (0–60)	24.90 (11.44)	17.16 (9.48)	23.94 (15.22)	18.15 (12.57)	0.51 (0.00)	9.97*** (0.08)	3.92* (0.03)	0.28 (0.00)	3.60 (0.03)	0.02 (0.00)	6.99** (0.06)
WM performance retest (0–60)	22.77 (11.43)	19.42 (10.91)	29.00 (15.10)	19.22 (11.16)							
IES-R Total (0–88; 0.91)	8.23 (8.24)	11.03 (12.35)	4.52 (6.08)	7.04 (11.99)	4.49* (0.04)	2.15 (0.02)	–	0.01 (0.00)	–	–	–
IES-R Intrusion (0–32; 0.85)	3.06 (3.41)	3.48 (4.13)	2.06 (3.27)	3.22 (4.93)	0.76 (0.01)	1.19 (0.01)	–	0.26 (0.00)	–	–	–
IES-R Avoidance (0–32; 0.80)	4.48 (4.63)	5.97 (6.64)	2.16 (2.81)	2.70 (4.54)	9.89*** (0.08)	1.30 (0.01)	–	0.28 (0.00)	–	–	–
ERRI Intrusive lab session (0–3; 0.89)	0.75 (0.64)	0.95 (0.68)	0.61 (0.60)	1.07 (0.67)	0.60 (0.01)	3.63 (0.03)	41.12**** (0.26)	0.82 (0.01)	1.27 (0.01)	8.68*** (0.07)	0.69 (0.01)
ERRI Intrusive 24-h (0–3; 0.88)	0.57 (0.50)	0.55 (0.45)	0.41 (0.48)	0.45 (0.57)							
ERRI Deliberate lab session (0–3; 0.87)	0.51 (0.43)	0.79 (0.61)	0.27 (0.43)	0.74 (0.55)	1.96 (0.02)	8.43*** (0.07)	25.77**** (0.18)	0.24 (0.00)	0.68 (0.01)	12.74**** (0.10)	1.61 (0.01)
ERRI Deliberate 24-h (0–3; 0.88)	0.37 (0.50)	0.48 (0.39)	0.29 (0.53)	0.39 (0.48)							

^*^ Degrees of freedom for Fisher’s F statistics = 1, 116. *p < 0.05, **p < 0.01, ***p < 0.005, ****p < 0.001.

**FIGURE 1:**
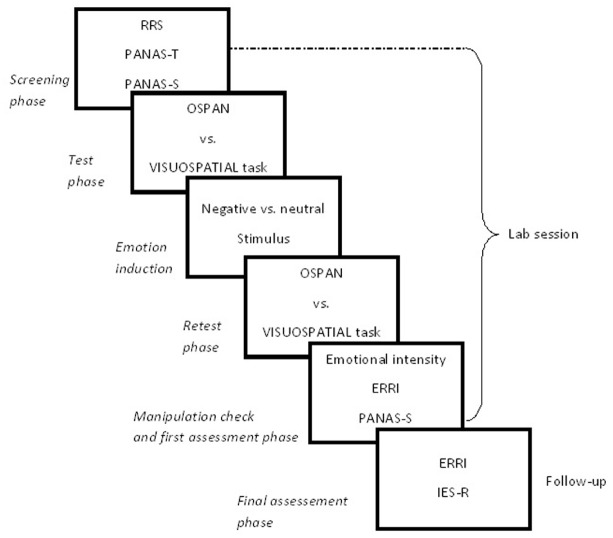
**Flowchart of the procedure, measures, and materials employed in Experiment 1 and 2.** The procedure for both experiments included: (1) a screening phase in which participants completed RRS, PANAS-T, and PANAS-S; (2) a test phase on WM capacity in which participants completed either the OSPAN or a Visuospatial analog task; (3) an emotion induction phase in which either a narrative excerpt (Experiment 1) or a video clip (Experiment 2) was administered to participants; (4) a re-test phase on participants’ WM capacity (OSPAN vs. Visuospatial task); (5) a manipulation check and first assessment phase in which participants answered questions concerning their emotional experience, ERRI and PANAS-S; and (6) a final assessment after 24 h in which a new ERRI and IES-R were administered to participants.

The procedure included: (1) a screening phase; (2) a test phase on participants’ WM capacity; (3) an emotion induction phase; (4) a re-test phase on WM capacity; (5) a manipulation check and first assessment phase; and (6) a final assessment after 24 h. Figure 1 display a workflow of the procedure, along with the measures and materials adopted in the experiment.

***Screening phase***

On arriving at the lab, participants were preliminarily administered the Ruminative Response Scale (RRS; Treynor et al., 2003) and the Positive and Negative Affect Schedule-Trait and State (PANAS-T and -S; Watson et al., 1988; Terracciano et al., 2003) in order to exclude confounding due to a depressive rumination style or affectivity disposition.

***Test phase***

Participants were seated in a quiet room in front of a computer and the experimental session started with instructions presented on the screen using SuperLab Pro v4.0 software. Participants were randomly assigned to one of two conditions of WM task (Verbal vs. Visuospatial). The task consisted of either a verbal assignment (i.e., the Operation-word memory span test-OSPAN; Turner and Engle, 1989) or an analog visuospatial task. Both tasks were employed as measures of central executive functioning among healthy individuals (Miyake et al., 2000), able to capture variations in sustaining attention and resisting interference (Rosen and Engle, 1998).

***Emotion induction***

Participants were randomly assigned to one of two emotional conditions and requested to read a two page excerpt of either negative emotional or neutral valence. The negative condition consisted of an excerpt from the novel *The Wind-Up Bird Chronicle* by Murakami Haruki, containing a realistic description of the torture of a prisoner of war by Mongolian soldiers. The neutral condition consisted of a description of the history and rules of the *Game of the Goose*, taken from Wikipedia.

***Retest phase***

Immediately after reading the excerpt, participants performed a new WM task session (with different stimuli but within the same procedure of the test phase).

***Manipulation check and first assessment phase***

Participants provided a short description of the excerpt and answered questions concerning its emotional intensity and PANAS-S. They also completed the ERRI (Cann et al., 2011), which evaluates two dimensions corresponding to intrusive and deliberate forms of rumination^1^.

***Final assessment phase***

Once at home, participants received a questionnaire by email containing the IES-R (Weiss and Marmar, 1996), specifically intended to measure the amount of protracted distress associated with a specific event and ERRI. They were instructed to fill in the questionnaire exactly 24 h after their participation in the lab session and to send it back by email. All participants returned the questionnaire on time. Neither data exclusion nor additional manipulations were run. 1 week after the lab session participants were debriefed individually or by email.

#### Measures and Materials

The scales adopted at each phase of the procedure are described in this section. For all measures, the ranges and Cronbach’s alphas are reported in Table 1.

***Screening phase***

Ruminative Response Scale (Treynor et al., 2003). The scale requires participants to rate on twenty-two 4-point scales (1 = never; 4 = always) how often they engage in ruminative responses following sad or depressive feelings. Items were averaged into three composite indices of Brooding, Depression, and Reflection, following the norms of the Italian validation.

Positive and Negative Affect Schedule-Trait (Watson et al., 1988; Terracciano et al., 2003). The scale requires participants to rate on twenty 5-point items (0 = not at all; 5 = completely) how they generally feel along two dimensions, corresponding to Positive Affect (PA-T) and Negative Affect (NA-T). PA-T reflects the extent to which a person generally feels enthusiastic, excited, active, and determined. NA-T reflects a general subjective distress and aversive affect.

Positive and Negative Affect Schedule-State (Watson et al., 1988; Terracciano et al., 2003). Participants were requested to rate on twenty 5-point scales (0 = not at all; 5 = completely) how they felt in that precise moment along two dimensions, corresponding to Positive Affect (PA-S), and Negative Affect (NA-S).

***Test and retest phases***

Verbal task. We administered participants the OSPAN, adapted into Italian from the original procedure by Turner and Engle (1989). Participants read on a computer screen 60 strings each composed of a simple mathematical equation [i.e., (3 × 4) – 5 = 6] followed by a 2-syllabe word (i.e., water). Participants had to press a different key depending on whether they rated it as correct or incorrect. At the same time they were requested to read out the word presented after the equation. The experimental phase was preceded by three practice trials. In the experimental phase, the strings were grouped into three blocks of five trials. The trial size varied from two to six strings. The order of trial sizes was randomized so that participants were not able to predict the number of strings in each trial. After each trial participants were requested to remember and write down as many words as possible from the strings. If the participant correctly identified the right and wrong equations in a given trial, at the same time, remembering all words in the trial in the same order of presentation, a score corresponding to the number of strings included in the trial was assigned to that participant. It follows that the total Verbal scores in the present study ranged from 0 to 60.

Visuospatial task. The task was modeled on the OSPAN but instead of verbal stimuli (equations and words) it includes visuospatial elements (i. e., Japanese ideograms and their spatial locations on the computer screen). Sixty ideograms were individually presented on the screen for 1000 ms, located in one of the four quadrants in which the space was ideally divided. The presentation of each ideogram was followed by a blank screen for 300 ms. An ideogram was then presented in the middle of the screen and participants were told to press a different key depending on whether they rated it as the same ideogram presented before or as a new one. The experimental phase was preceded by three practice trials. In the experimental phase, ideograms were grouped in three blocks of trials including two to six elements. The order of trial sizes was randomized so that participants could not predict the number of items in each trial. After each trial participants were presented with a blank screen divided into four quadrants by black margins, and had to use the computer mouse to indicate the portions of space occupied by the ideograms just identified in the same order of presentation. If the participant correctly recognized all ideograms in a given trial and remembered the locations of the stimuli in the trial in the same order of presentation, a score corresponding to the number of ideograms of the trial was assigned to that participant. It follows that the total Visuospatial scores in the present study ranged from 0 to 60. A pilot study was run to evaluate the validity of the task as a measure of WM capacity applied to visuospatial stimuli, with *N* = 17. The Visuospatial score was found to be correlated with the index of the Corsi block-tapping test (Orsini et al., 1987; Kessels et al., 2000) as a measure of visual memory span (Spearman’s rho = 0.59, *p* < 0.05), and with indices obtained from the Random Number Generation task (Ginsburg and Karpiuk, 1994), such as RNG (Random Number Generation Index—a measure of randomness of sequences; Spearman’s rho = 0.60, *p* < 0.05), NSQ (Null-Score Quotient—a measure of permutations that do not occur in the subject’s sequence; Spearman’s rho = 0.55, *p* < 0.05), and A (Adjacency—a measure of adjacent items in a sequence; Spearman’s rho = 0.50, *p* < 0.05; Towse and Neil, 1998). It should be noted that these three RNG indices were shown to be associated with the inhibition component of executive functioning (Miyake et al., 2000).

***Manipulation check, first and final assessment phases***

Emotional intensity. Participants were first requested to provide a short description of the material presented during the experimental phase and then rate the intensity of the emotion experienced during the reading of an 11-point scale (0 = not at all; 10 = the highest emotion in life).

Impact of Event Scale-Revised (Weiss and Marmar, 1996). An adapted version of the IES-R was administered which assessed the participants’ responses to exposure to emotional material 24 h after their participation in the lab session. The IES-R comprised twenty-two 5-point scales, ranging from 0 (= not at all) to 4 (= very much). All items were summed up into a total IES-R score. Additionally, two sub dimensions of Intrusion and Avoidance were computed from two subsets of items of the scale^2^.

Event Related Rumination Inventory (Cann et al., 2011). Rumination was assessed through a self-report inventory composed of twenty 4-point scales (1 = never; 4 = often) evaluating two dimensions corresponding to Intrusive Rumination (the persistence of unwanted disturbing thoughts related to an emotional experience) and Deliberate Rumination (i.e., the tendency to engage in intentional elaborations concerning an emotional experience). Items of the ERRI were adapted to the emotional material (i. e., the novel excerpt) presented in the experiment.

### Results

#### Screening Analysis

To assess whether participants differed in their tendency toward a depressive rumination style and affectivity disposition, a set of factorial ANOVAs was run on the three indices of RRS, with Emotional valence (Negative vs. Neutral) and Task modality (Verbal vs. Visuospatial) as independent variables. Neither the main nor interaction effects reached the significance level (see top panel Table 1). Similar factorial ANOVAs were run on the indices of PANAS-T, but no effects were observed on either positive or negative scores (see top panel Table 1). It follows that no participants in the 2 × 2 cells of the design differed in their tendency toward a depressive rumination style and affectivity disposition.

#### Manipulation Checks

In order to exclude individual differences in emotional states before the experimental manipulation and to verify the effect of the emotional induction, PANAS-S (PA-S and NA-S) scores were analyzed through 2 × 2 × 2 mixed design ANOVAs, with Emotional valence (Negative vs. Neutral) and Task modality (Verbal vs. Visuospatial) as between subjects factors and Test–retest as a within subjects factor. Table 1 displays the outcomes of these analyses. Focusing on the interaction effects of Emotional valence by Test–retest [*F*(1, 116) = 4.01, *p* < 0.05 partial η^2^ = 0.03 and *F*(1, 116) = 12.63, *p* < 0.001 partial η^2^ = 0.10, for PA-S and NA-S respectively], no significant differences were observed in participants’ emotional states before the experimental induction [*F*s (1, 118) < 0.04, *n.s.*, partial η^2^ = 0.00], but, at retest, PA-S scores were lower in the negative condition than in the neutral condition [*F*(1, 118) = 4.44, *p* < 0.05, partial η^2^ = 0.04; *M*_Negative_ = 2.00, SD = 0.81; *M*_Neutral_ = 2.33, SD = 0.87], and NA-S scores were higher in the negative condition than in the neutral condition [*F*(1, 118) = 12.23, *p* < 0.001, partial η^2^ = 0.09; *M*_Negative_ = 0.75, SD = 0.76; *M*_Neutral_ = 0.31, SD = 0.57]. These findings confirmed that the experimental manipulation of participants’ emotional states was successful. Reading the negative excerpt had the effect of increasing participants’ negative emotional states and decreasing positive emotional states.

The index of emotional intensity was analyzed in a 2 × 2 between subject design ANOVA, with Emotional valence (Negative vs. Neutral) and Task modality (Verbal vs. Visuospatial), as between subjects factors (see Table 1). The only significant effect was that of Emotional valence [*F*(1, 116) = 66.18, *p* < 0.001 partial η^2^ = 0.36], with individuals in the negative condition scoring significantly higher than individuals in the neutral condition (*M*_Negative_ = 4.98, SD = 3.32; *N*_Neutral_ = 0.91, SD = 1.87). Neither the other main nor the interaction effects were found to be significant. These findings provided further confirmation for the efficacy of our manipulation.

#### Analyses of WM Performance Indices

**FIGURE 2:**
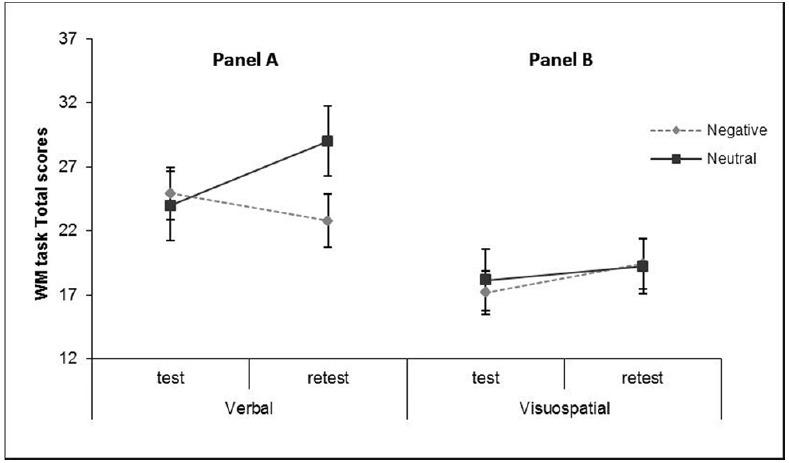
**Three-way interaction effects of Emotional valence by Task modality by Test–retest on the WM performance scores, Experiment 1.** The three-way interaction effects of Emotional valence by Task modality by Test–retest on the WM performance scores was found to be significant. **(A)** Simple effect analyses confirmed that for participants assigned to the Verbal session the performance scores significantly increased over time in the neutral condition, but remained stable in the emotional condition. **(B)** the Visuospatial performance remained substantially stable across emotional conditions over time. These findings indicate that the visual performance was generally unaffected by practice whereas the verbal performance was influenced by participants’ practice only in the neutral condition. Error bars represent ± 1 SE.

To assess whether the exposure to verbal emotional material affected the ability of participants to accomplish a verbal vs. visuospatial WM task, Total scores of the WM performance were entered in a 2 × 2 × 2 mixed design ANOVA with Emotional valence (Negative vs. Neutral) and Task modality (Verbal vs. Visuospatial) as between subjects factors and Test–retest as a within subjects factor. As shown in Table 1, the main effects of the Task modality and Test–retest factors were found to be significant [*F*(1, 116) = 9.97, *p* < 0.005 partial η^2^ = 0.08, and *F*(1, 116) = 3.92, *p* < 0.05 partial η^2^ = 0.03, respectively]. Participants’ Visuospatial WM performances were generally poorer than for the Verbal performance (*M*_Verbal_ = 25.15, SD = 12.85; *M*_Visuospatial_ = 18.47, SD = 9.81), and generally improved at retest (*M*_Test_ = 21.13, SD = 12.69; *M*_Retest_ = 22.72, SD = 12.79) as an effect of participants becoming more proficient with practice. The three-way interaction was found to be significant [*F*(1, 116) = 6.99, *p* < 0.01 partial η^2^ = 0.06, see Figure 2]. Simple effect analyses confirmed that a practice effect occurred for participants assigned to the verbal session in the neutral condition [*F*(1, 118) = 10.56, *p* < 0.005, partial η^2^ = 0.08], not in the emotional condition [*F*(1, 118) = 1.87, *n.s.*, partial η^2^ = 0.02; panel A], however, the performance of participants completing the visuospatial task remained substantially stable over time [*F*s (1, 118) < 2.00, *n.s.*, partial η^2^ < 0.02; panel B]. These findings indicate that visuospatial performance was generally unaffected by practice whereas verbal performance was only influenced by participants’ practice in the neutral condition. To exclude the possibility that the difficulty of the visuospatial task be a confounding effect in these analyses, we standardized the WM scores within each Task modality condition, and ran a new ANOVA on these standardized scores. We obtained the same pattern of results for the three-way interaction, thus ruling out a possible effect of a different level of task difficulty.

In sum, in conformity with our prediction (1), we observed that individuals exposed to neutral material improved their WM performance with practice, while individuals exposed to emotional material did not improve their performance at retest. These findings indicate that a failure occurred in performing the cognitive task when it shared the same (verbal) modality of the emotional material to which the individual was exposed.

#### Analyses of IES-R and ERRI

We predicted that intrusions concerning verbal emotional material were only affected by the emotional valence of the original material and were independent upon the modality (verbal vs. visuospatial) of the concurrent WM task. To test this hypothesis, a set of 2 × 2 ANOVAs were run on the IES-R scores (Total, Intrusion, Avoidance) with Emotional valence (Negative vs. Neutral) and Task modality (Verbal vs. Visuospatial) as between subjects factors (see Table 1). The main effect of Emotional valence was significant for the Total IES-R scores [*F*(1, 116) = 4.49, *p* < 0.05 partial η^2^ = 0.04; *M*_Negative_ = 9.63, SD = 10.51; *M*_Neutral_ = 5.69, SD = 9.30], and for the Avoidance Index [*F*(1, 116) = 9.89, *p* < 0.005 partial η^2^ = 0.08; *M*_Negative_ = 5.23, SD = 5.72; *M*_Neutral_ = 2.41, SD = 3.69]. Neither other main nor interaction effects reached the significance level, so that intrusions appeared to be affected only by the emotional valence of the excerpt.

**FIGURE 3:**
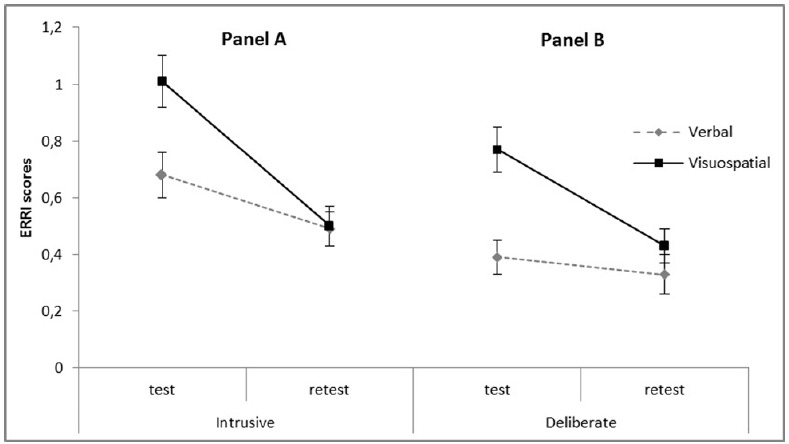
**Two-way interaction effects of Task modality by Test–retest on the ERRI scores, Experiment 1. (A)** The two-way interaction effects of Task modality by Test–retest on Intrusive index was significant in that, at the test, participants assigned to the Visuospatial condition reported higher scores of Intrusive rumination as compared with participants assigned to the Verbal condition. This significant divergence vanished at retest, although a significant decrease over time was observed for both conditions of task modality. **(B)** The two-way interaction effects of Task modality by Test–retest on Deliberate index was significant. Analyses of the simple effects demonstrated that, at the test, rates of Deliberate rumination were significantly higher in the Visuospatial condition as compared with the Verbal, but significantly dropped over time in the Visuospatial task condition, while remaining substantially stable in the Verbal condition. The significant divergence observed at test vanished at retest. Error bars represent ± 1 SE.

As regards rumination, we predicted that, following the exposure to verbal emotional material, individuals would engage in long-term rumination to a higher extent if they were concurrently requested to perform a verbal than a visuospatial task. To test this hypothesis, two 2 × 2 × 2 mixed design ANOVAs were run on the ERRI scores with Emotional valence (Negative vs. Neutral) and Task modality (Verbal vs. Visuospatial) as between subjects factors and Follow-up (at the end of the lab session vs. at a 24-h delay) as a within subjects factor (see Table 1, bottom rows). The analysis on the Intrusive index resulted in a significant main effect of Follow-up due to a general decrease in the rates of intrusive rumination over time [*F*(1, 116) = 41.12, *p* < 0.001 partial η^2^ = 0.26; *M*_lab_ = 0.84, SD = 0.66; *M*_24 h_ = 0.50, SD = 0.50]. An interaction effect of Task modality by Follow-up was also observed [*F*(1, 116) = 8.68, *p* < 0.005 partial η^2^ = 0.07] in that, at the end of the lab session, participants assigned to the visuospatial condition reported higher scores of Intrusive rumination as compared with participants assigned to the verbal condition [*F*(1, 118) = 7.84, *p* < 0.01, partial η^2^ = 0.06], this significant divergence vanished after 24 h [*F*(1, 118) = 0.02, *n.s.*, partial η^2^ = 0.00; see Figure 3, panel A]. Neither other main nor interaction effects reached the significance level. The analyses on the Deliberate scores resulted in significant main effects of Task modality [*F*(1, 116) = 8.43, *p* < 0.005 partial η^2^ = 0.07; *M*_Verbal_ = 0.36, SD = 0.44; *M*_Visuospatial_ = 0.60, SD = 0.45], Follow-up [*F*(1, 116) = 25.77, *p* < 0.001 partial η^2^ = 0.18; *M*_lab_ = 0.57, SD = 0.55; *M*_24 h_ = 0.38, SD = 0.48], and an interaction of Task modality by Follow-up [*F*(1, 116) = 12.74, *p* < 0.001 partial η^2^ = 0.10], neither other main nor interaction effects reached the significance level. To qualify the interaction, analyses of the simple effects demonstrated that, at the end of the lab session, rates of deliberate rumination were significantly higher in the visuospatial condition as compared with the verbal condition [*F*(1, 118) = 16.21, *p* < 0.001, partial η^2^ = 0.12], the significant divergence observed at the lab session vanished after 24 h [*F*(1, 118) = 1.41, *n.s.*, partial η^2^ = 0.01; see Figure 3, panel B]^3^.

To sum up, findings concerning the indices of intrusions and rumination partly contradict prediction (2): For individuals exposed to verbal emotional material, intrusions were only affected by the emotional valence of the original material (Hackmann et al., 2004; Curci et al., 2013), but rates of long-term rumination were higher when the concurrent WM task had a visuospatial modality.

### Discussion

The results from Experiment 1 supported some of our hypotheses. The test–retest design resulted in a practice effect on WM performance only for participants assigned to the neutral emotional valence condition and Verbal task. This means that reading an emotional excerpt represents a significant disturbance in cognitive performance when it shares the same (verbal) sensory modality, but is ineffective when the task to be accomplished is visuospatial. A reason for this effect might be that the visuospatial task was experienced as significantly more difficult than the verbal task, thus depleting the individual’s WM resources to a significantly higher extent. The effect of valence was observed to be significant on the IES-R total and Avoidance scores, in conformity with our previous study (Curci et al., 2013). However, as regards rumination, it should be noted that in Experiment 1 neither the emotional valence effect not its interactions with other factors of the design were observed as being significant, so it seems that simply interrupting a visuospatial performance with either negative or neutral material resulted in an increase in ruminative thoughts. These results led us to carry out further research work by presenting participants with a negative vs. neutral video clip instead of a novel excerpt (see Holmes and Bourne, 2008), as stimulus material. This choice derived from the assumption that visual material is more effective in triggering an emotional reaction than simply reading a text taken from a novel, strengthened by evidence from Experiment 1 which demonstrated a prevalence of the visuospatial modality upon verbal processing.

## Experiment 2

### Materials and Methods

#### Participants and Design

A total of 163 participants (86.5% women; *M*_age_ = 19.28, SD = 0.83) were involved in the study. The 2 × 2 between subjects design and the dependent measures of Experiment 2 were identical to those in Experiment 1.

#### Procedure, Measures, and Materials

Participants were recruited during a Psychology class and requested to take part in an experimental study. A total of 168 students agreed to participate, but five of these abandoned the experimental session before completing it. The procedure, measures, and materials were identical to those used in Experiment 1 (see Figure 1), except for the emotional stimuli adopted to manipulate participants’ emotional state. In the present experiment we used a 2-min. video clip of negative vs. neutral content and participants were randomly assigned to one of two emotional valence conditions. The negative condition consisted in a realistic scene of the medieval torture of face flaying and the neutral condition consisted in a procedure for making origami. Participants were warned that the clips might have a shocking content and those who were unwilling to continue their participation (*n* = 5) were thanked and dismissed. The distribution of the sample across conditions is reported in the heading row of Table 2. In the same table we included ranges and Cronbach’s alphas for all measures adopted in this experiment.

**TABLE 2:** **ANOVAs on the measures of screening and experimental phases, Experiment 2**.

									***F* (partial **η^2^**)**						
**Measures (min-max; Cronbach’s alpha)**	**Negative *M* (SD)**	**Negative *M* (SD)**	**Neutral *M* (SD)**	**Neutral *M* (SD)**	**Emotional valence**	**Task**	**Test–retest**				
	**Verbal (n = 41)**	**Visuospatial (n = 41)**	**Verbal (n = 42)**	**Visuospatial (n = 39)**	**(a)**	**(b)**	**(c)**	**(a × b)**	**(a × c)**	**(b × c)**	**(a × b × c)**
**Screening phase**										
RRS-Brooding (1–4; 0.76)	2.49 (0.48)	2.32 (0.49)	2.39 (0.42)	2.42 (0.52)	0.00 (0.00)	0.91 (0.01)	–	1.83 (0.01)	–	–	–
RRS-Depression (1–4; 0.64)	2.31 (0.58)	2.29 (0.49)	2.31 (0.53)	2.18 (0.47)	0.43 (0.00)	0.87 (0.01)	–	0.51 (0.00)	–	–	–
RRS-Reflection (1–4; 59)	2.36 (0.55)	2.15 (0.59)	2.27 (0.55)	2.23 (0.56)	0.00 (0.00)	1.86 (0.01)	–	0.92 (0.01)	–	–	–
PA-T (0–4; 0.71)	2.69 (0.45)	2.72 (0.44)	2.65 (0.44)	2.70 (0.42)	0.18 (0.00)	0.31 (0.00)	–	0.03 (0.00)	–	–	–
NA-T (0–4; 0.86)	1.43 (0.67)	1.50 (0.67)	1.40 (0.66)	1.35 (0.75)	0.65 (0.00)	0.01 (0.00)	–	0.33 (0.00)	–	–	–
**Experimental phase**										
PA-S test (0–4; 0.84)	2.65 (0.55)	2.62 (0.59)	2.78 (0.58)	2.77 (0.57)	6.20* (0.04)	0.02 (0.00)	124.50**** (0.44)	.12 (0.00)	4.35* (0.03)	0.03 (0.00)	0.25 (0.00)
PA-S retest (0–4; 0.90)	2.05 (0.78)	1.98 (0.78)	2.33 (0.76)	2.38 (0.79)							
NA-S test (0–4; 0.88)	0.62 (0.60)	0.72 (0.65)	0.66 (0.58)	0.54 (0.55)	13.97**** (0.08)	0.10 (0.00)	1.27 (0.01)	0.62 (0.00)	32.11**** (0.17)	0.33 (0.00)	1.12 (0.01)
NA-S retest (0–4; 0.90)	0.88 (0.73)	0.85 (0.69)	0.35 (0.42)	0.28 (0.42)							
Emotional intensity (0–10)	5.55 (2.75)	4.98 (2.61)	1.29 (2.13)	1.23 (1.86)	114.54**** (0.43)	0.71 (0.01)	–	0.49 (0.00)	–	–	–
WM performance test (0–60)	24.68 (14.31)	16.68 (8.38)	23.76 (13.34)	16.64 (10.60)	0.52 (0.00)	19.75*** (0.11)	7.83** (0.05)	0.00 (0.00)	5.76* (0.04)	0.29 (0.00)	0.59 (0.00)
WM performance retest (0–60)	24.80 (14.95)	17.15 (10.55)	28.55 (13.42)	19.51 (11.66)							
IES-R Total (0–88; 0.90)	14.68 (9.40)	17.63 (13.14)	4.62 (6.07)	6.18 (7.64)	52.91**** (0.25)	2.33 (0.01)	–	0.22 (0.00)	–	–	–
IES-R Intrusion (0–32; 0.80)	5.46 (3.72)	6.68 (4.95)	2.57 (3.19)	3.03 (3.47)	28.80**** (0.15)	1.88 (0.01)	–	0.39 (0.00)	–	–	–
IES-R Avoidance (0–32; 0.79)	7.17 (4.92)	8.20 (5.70)	1.62 (2.67)	2.18 (3.06)	74.28**** (0.32)	1.39 (0.01)	–	0.12 (0.00)	–	–	–
ERRI Intrusive lab session (0–3; 0.91)	1.22 (0.66)	1.18 (0.73)	0.72 (0.65)	0.70 (0.66)	34.85**** (0.18)	0.00 (0.00)	19.61**** (0.11)	0.06 (0.00)	0.03 (0.00)	0.31 (0.07)	0.06 (0.00)
ERRI Intrusive 24-h (0–3; 0.90)	0.97 (0.50)	0.96 (0.60)	0.46 (0.53)	0.52 (0.52)							
ERRI Deliberate lab session (0–3; 0.78)	0.78 (0.49)	0.82 (0.52)	0.49 (0.34)	0.52 (0.44)	21.44**** (0.12)	0.49 (0.00)	12.14**** (0.07)	0.01 (0.00)	0.16 (0.00)	0.03 (0.00)	0.05 (0.00)
ERRI Deliberate 24–h (0–3; 0.78)	0.66 (0.41)	0.69 (0.47)	0.38 (0.36)	0.44 (0.41)							

^*^ Degrees of freedom for Fisher’s F statistics = 1, 159. *p < 0.05, **p < 0.01, ***p < 0.005, ****p < 0.001.

### Results

#### Screening Analysis

To assess whether participants differed in their tendency toward a depressive rumination style and affectivity disposition, a set of factorial ANOVAs was run on RRS and PANAS-T indices with Emotional valence (Negative vs. Neutral) and Task modality (Verbal vs. Visuospatial) as independent variables. Table 2, top panel, displays the outcomes of these analyses, showing that the sample of participants was found homogeneous regarding its tendency toward depressive rumination and affectivity disposition since neither the main nor interaction effects reached the significance level on any RRS or PANAS-T scale.

#### Manipulation Checks

In order to exclude individual differences in emotional states before the experimental manipulation and to verify the effect of the emotional induction, the PA-S and NA-S scores were entered in a 2 × 2 × 2 mixed design ANOVA, with Emotional valence (Negative vs. Neutral) and Task modality (Verbal vs. Visuospatial) as between subjects factors and Test–retest as a within subjects factor. Results of these analyses are displayed in Table 2, and special attention has to be given to the interaction effect of Emotional valence by Test–retest [*F*(1, 159) = 4.35, *p* < 0.05 partial η^2^ = 0.03, and *F*(1, 159) = 32.11, *p* < 0.001 partial η^2^ = 0.17, for PA-S and NA-S respectively]. The simple effect analyses run to qualify these interactions showed that, as in the case of Experiment 1, no significant differences were found in the emotional state of the 2 × 2 groups of participants before the experimental induction [*F*s (1, 161) < 2.60, *n.s.*, partial η^2^ < 0.03], but, at retest, PA-S scores were lower in the negative condition than in the neutral condition [*F*(1, 161) = 7.72, *p* < 0.01, partial η^2^ = 0.05; *M*_Negative_ = 2.01, SD = 0.78; *M*_Neutral_ = 2.35, SD = 0.77], and NA-S scores were higher in the negative condition than in the neutral condition [*F*(1, 161) = 35.90, *p* < 0.001, partial η^2^ = 0.18; *M*_Negative_ = 0.86, SD = 0.71; *M*_Neutral_ = 0.32, SD = 0.42]. Taken together, these findings demonstrated that the experimental manipulation of participants’ emotional states was successful. Exposure to the emotional film had the effect of increasing the negative emotional states and reducing the positive emotional states in our sample.

The index of Emotional intensity was analyzed in a 2 × 2 between subject design ANOVA with Emotional valence (Negative vs. Neutral) and Task modality (Verbal vs. Visuospatial) as between subjects factors (see Table 2). The only significant effect was of Emotional valence [*F*(1, 159) = 114.54, *p* < 0.001 partial η^2^ = 0.43], with individuals in the negative condition scoring significantly higher than individuals in the neutral condition (*M*_Negative_ = 5.26, SD = 2.68; *N*_Neutral_ = 1.26, SD = 1.99). Neither the other main nor the interaction effects approached significance, thus confirming the efficacy of our manipulation.

#### Analyses of the WM Performance Indices

**FIGURE 4:**
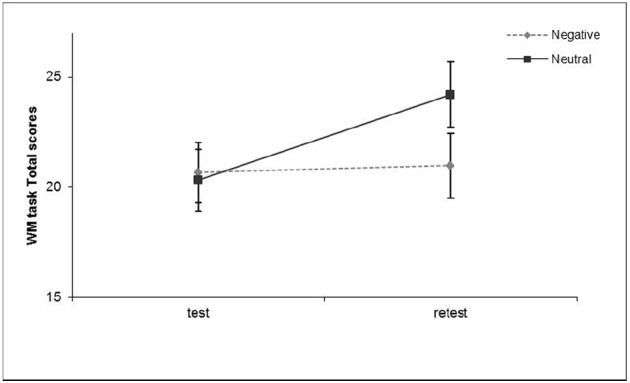
**Two-way interaction effects of Emotional valence by Test–retest on the WM performance scores, Experiment 2.** The two-way interaction of Emotional valence by Test–retest on the executive performance scores was significant, in that practice improved participants’ performance after the exposure to the neutral film, but was ineffective in the negative emotional condition. These findings demonstrates that the exposure to an emotional film hinders the practice effects upon the WM performance, independent on the sensory modality of the task. Error bars represent ± 1 SE.

To assess whether the exposure to visual emotional material affected the ability of participants to accomplish a verbal vs. visuospatial WM task, a 2 × 2 × 2 mixed design ANOVA was run on the total WM scores with Emotional valence (Negative vs. Neutral) and Task modality (Verbal vs. Visuospatial) as between subjects factors and Test–retest as a within subjects factor. The main effects of the Task modality and the Test–retest factors were found to be significant [respectively, *F*(1, 159) = 19.75, *p* < 0.005 partial η^2^ = 0.11, and *F*(1, 159) = 7.83, *p* < 0.01 partial η^2^ = 0.05; see Table 2]. Participants’ performances were generally poorer for the visuospatial task than for the verbal (*M*_Verbal_ = 25.46, SD = 12.99; *M*_Visuospatial_ = 17.48, SD = 9.39) and generally improved at retest (*M*_Test_ = 20.51, SD = 12.40; *M*_Retest_ = 22.58, SD = 13.43) as an effect of participants’ practice. The two-way interaction of Emotional valence by Test–retest was significant [*F*(1, 159) = 5.76, *p* < 0.05 partial η^2^ = 0.04], in that practice improved participants’ WM performance after the exposure to the neutral film [*F*(1, 161) = 13.78, *p* < 0.001, partial η^2^ = 0.08] but was ineffective in the negative emotional condition [*F*(1, 161) = 0.08, *n.s.*, partial η^2^ = 0.00; see Figure 4]. Neither other main nor interaction effects reached the significance level. Taken together, these findings demonstrate that exposure to an emotional film hinders the effects of practice on WM performance, no matter the sensory modality of the task. This conclusion contradicts our prediction (1), since the processing of the visual content of an emotional experience seems to prevail in competition with any concomitant cognitive activity and not only with the visuospatial task.

#### Analyses of IES-R and ERRI

In this section we report the outcomes of the analyses run to verify that, for individuals exposed to visual emotional material, intrusions and rumination will only be influenced by the emotional valence of the stimulus material and will persist over time regardless of the modality (verbal vs. visuospatial) of the concurrent WM task. Table 2 displays the outcomes of a set of 2 × 2 ANOVAs run on the IES-R scores (Total, Intrusion, Avoidance) with Emotional valence (Negative vs. Neutral) and Task modality (Verbal vs. Visuospatial) as between subjects factors (see Table 2, bottom panels). The main effect of Emotional valence was significant for all three indices [*F*s (1, 159) > 28.79, *p* < 0.001 partial η^2^ > 0.14], i.e., the Total IES-R (*M*_Negative_ = 16.15, SD = 11.45; *M*_Neutral_ = 5.37, SD = 6.88), Intrusion (*M*_Negative_ = 6.07, SD = 4.40; *M*_Neutral_ = 2.79, SD = 3.32) and Avoidance index (*M*_Negative_ = 7.68, SD = 5.32; *M*_Neutral_ = 1.89, SD = 2.86). Neither other main nor interaction effects reached the significance level.

Finally, the last rows of Table 2 report the results of two 2 × 2 × 2 mixed design ANOVAs on the ERRI scores (Intrusive and Deliberate), with Emotional valence (Negative vs. Neutral) and Task modality (Verbal vs. Visuospatial) as between subjects factors and Follow-up (at the end of the lab session vs. at a 24-h delay) as a within subjects factor. Analysis of the Intrusive index resulted in significant main effects of Emotional valence [*F*(1, 159) = 34.85, *p* < 0.001, partial η^2^ = 0.18] and Follow-up [*F*(1, 159) = 19.61, *p* < 0.001, partial η^2^ = 0.11]. The rates of intrusive rumination were higher in the emotional condition as compared with the neutral condition (*M*_Negative_ = 1.08, SD = 0.54; *M*_Neutral_ = 0.60, SD = 0.49) and declined over time for participants in all conditions of the design (*M*_lab_ = 0.96, SD = 0.71; *M*_24 h_ = 0.73, SD = 0.59). Similarly, the analyses on the Deliberate scores were found to be significantly higher in the emotional condition as compared with the neutral condition [*F*(1, 159) = 21.44, *p* < 0.001, partial η^2^ = 0.12; *M*_Negative_ = 0.74, SD = 0.42; *M*_Neutral_ = 0.46, SD = 0.34] and significantly declined over time [*F*(1, 159) = 12.14, *p* < 0.001, partial η^2^ = 0.07; *M*_lab_ = 0.65, SD = 0.47; *M*_24 h_ = 0.54, SD = 0.43]^4^.

Taken together, these results confirm our hypothesis (3), in that, for individuals exposed to visual emotional material, intrusions and rumination persisted over time regardless of the WM task modality (verbal vs. visuospatial). Indeed, these findings indicate a prevalence of visual emotional material in sustaining post-emotional elaboration over time.

### Discussion

The results from Experiment 2 slightly differ from those of Experiment 1. Exposure to emotional material overloads the individual’s cognitive resources so that WM performance is hampered, no matter what the sensory modality of the task. In contrast, exposure to neutral material results in a practice effect for both verbal and visuospatial performance. As a counterpart of this process, intrusions and ruminative thoughts were formed to a higher extent for the emotional film than for the neutral one, with the task modality having no effect. Both intrusions and rumination seemed to develop as consequences of a cognitive overloading induced by the individual’s exposure to an emotional experience. Such an overloading appeared to be responsible for the faults in the performance immediately following the emotional experience and the long-term persistence of images and thoughts related to the same experience.

## General Discussion

In the two experiments described we aimed to demonstrate that following an emotional experience individuals develop intrusions and rumination which compete with the concomitant execution of other tasks, and prolong over time as a consequence of this competition. In Experiment 1, in accordance with our prediction (1), we observed that exposure to neutral material induced a practice effect upon WM performance, while this effect was not observed after exposure to emotional material. As regards the post-emotional processes, intrusions concerning verbal emotional material appeared to be only affected by the emotional valence, but, at odds with prediction (2), rates of long-term rumination were higher when the concurrent WM task has a visuospatial modality. In Experiment 2, in contradiction with prediction (1), the exposure to an emotional film appears to hinder practice effects on WM performance, regardless of the sensory modality of the task. By contrast, in line with our hypothesis (3), intrusions and rumination appeared to persist over time regardless of the concurrent modality of WM task (verbal vs. visuospatial). In sum, results from the two experiments, jointly considered, provide support for most of our predictions, and confirm that being exposed to emotional material results in an impairment of the individual’s cognitive performance in the short run. This impairment is dependent upon the modality (verbal vs. visuospatial) of the original material and the concurrent task. These findings need to be considered together with findings concerning intrusions and rumination.

Experiments 1 and 2 confirmed that intrusions only depend upon the emotional valence of the original experience. Individuals develop intrusions concerning their experiences as a consequence of the emotional impact of these experiences, regardless of the cognitive conflict induced with concomitant cognitive tasks. This result is consistent with our previous findings (Curci et al., 2013), but diverges from the results of studies by Holmes et al. (2009, 2010), who demonstrated that the pathological consequences of experiencing a trauma (i.e., intrusions and flashbacks) might be reduced through a kind of “cognitive vaccine” consisting of a visuospatial engagement of the individual in the computer game “Tetris.” A possible explanation for this discrepancy might be that Holmes et al. (2009, 2010) did not compare their emotional condition with exposure to neutral material but investigated the evolution of intrusions following a traumatic exposure over a 1-week period through diary reports and IES-R. Therefore, it is possible that the significant reduction in the amount of intrusions in the “Tetris” condition as compared with a no-task condition (Holmes et al., 2009, 2010), and a “Pub Quiz” condition was due to a natural decline of post-emotional elaboration over time (see Curci and Rimé, 2012), which was accelerated by (and not solely due to) a visuospatial cognitive intervention.

A different pattern of findings emerged for rumination. Rumination follows emotional experiences and tends to decline over time. In Experiment 1, the interaction between the verbal mode of the experience and the concurrent WM task resulted in an impairment of the immediate verbal performance and in a procrastination of ruminative processes only for individuals engaged in the visuospatial task. In Experiment 2, it is the visuospatial content of the emotional experience that prevails upon the concomitant verbal and visuospatial performance but rumination will develop in the long run regardless of the modality of task to which individuals are exposed. These results, although not completely in accordance with our expectations, provided confirmation for considering visuospatial processing as a more resource consuming process than any verbal engagement. According to the classical dual coding theory, the former requires both verbal and visual resources to be achieved, while the latter only relies upon verbal resources (Paivio, 1991). It follows that visuospatial processing can deflect an immediate performance when the cognitive conflict is unbearable as well as sustain ruminative processes over time. However, we should also consider that in Experiment 1 rumination was found to be unrelated with the emotional content of the experience and was only influenced by the mode of the concurrent WM task. This effect might be explained by the fact that an exposure to verbal contents *per se* requires a long-term cognitive elaboration, therefore, rumination flows as a cognitive processing of a learned experience (Experiment 1) but is amplified following emotional experiences of high intensity (Experiment 2).

An important element to consider in evaluating the present results is that we assessed rumination through the ERRI (Cann et al., 2011), an inventory specifically aimed at measuring two dimensions, corresponding to an intrusive dimension and a deliberate dimension. In Experiment 1, the rates of intrusive rumination decreased over time for both Verbal and Visuospatial conditions. On the other hand, the rates of deliberate rumination were found to decrease only for individuals completing the Visuospatial task and remained substantially stable for the Verbal task. This means that exposure to verbal material requires a continual cognitive elaboration preferably in the form of deliberate thinking. In contrast, intrusive thoughts ordinarily tend to weaken as do all any other symptoms of distress in non-pathological samples (Michael et al., 2005; Watkins, 2008). In Experiment 2, the emotional impact of visual material stimulates both forms of rumination which then declines, over time. From these findings, it follows that deliberate and intrusive post-emotional elaboration, although very similar phenomena—in our studies the Pearson’s coefficients of correlation ranged from 0.40 to 0.74—differ somewhat in their evolution (Ehlers and Clark, 2000; Santa Maria et al., 2012; Smets et al., 2012; Luo et al., 2013), and the nature of the triggering experience (verbal vs. visuospatial) intervenes in determining their course over time.

From a theoretical point of view, our data proved that integrating the modality approach (Hagenaars et al., 2010; Brewin and Burgess, 2014) with the distraction approach (Conway and Pleydell-Pearce, 2000; Conway et al., 2004; Gunter and Bodner, 2008; Krans et al., 2009) is possible and useful for the advancement of the understanding of the temporal evolution of emotional processes. Unlike studies referring to the modality hypothesis, we focused on the interaction between the modality of the cognitive task and the nature of the emotional material (verbal vs. visuospatial); beyond the distraction hypothesis, we stressed the role of visual elaboration which can have a prevalence in the cognitive balance between immediate and delayed processing. Overall, our findings encourage us to adopt an integrative theoretical perspective although more work is necessary to refine this approach and evaluate its implications in real life.

Some might object that our investigation of the interaction between the mode of cognitive task and the nature (verbal vs. visuospatial) of emotional experience is of limited interest since upsetting experiences mainly consist of visuospatial elements. However, real life is full of situations in which verbal features are crucial in determining an emotional reaction, such as a quarrel on the telephone or a turbulent email exchange. Social networks and phone chats overwhelm individuals with verbal information which is not always pleasurable in its content. The current studies demonstrate that when exposed to verbal emotional material individuals might benefit from running a concomitant verbal task in the long run, although immediate performance might be seriously hampered.

We acknowledge that the perspective adopted in running the current experiments did not take into account the role of individual differences in WM capacity. Pe et al. (2013), in their Study 2, showed that individual differences in the ability to update emotional information in WM moderates the efficacy of rumination on the experience of positive and negative emotions. Curci et al. (2013) showed that the individual’s availability of WM resources is a relevant predictor of ruminative thoughts both immediately after an ordinary emotional experience and at a 24-h delay. Further studies adopting the current paradigm might consider the role of individual differences in WM capacity prior to manipulation as an important moderator on the individual’s ability to control attention and develop intrusions and rumination in the long run.

Another issue deserving of further consideration concerns the process underlying the persistence of intrusion and rumination in a balance with a competing cognitive load. The recurrence of unprocessed material might be paralleled to a form of rebound effect (Gold and Wegner, 1995; Erber and Wegner, 1996). Erskine et al. (2007), suggested that it is possible to consider the relationship between rumination and suppression as bi-directional and self-reinforcing, given the frequent co-occurrence of the two phenomena in daily life. Given this perspective, we might hypothesize that people finding it difficult to suppress unwanted thoughts during concurrent activities may be more likely to develop rumination in the long run. The current experiments did not test this possibility and so future research work is necessary on this regard.

Finally, we acknowledge that the current investigation needs to be expanded with an appropriate control of the emotional intensity of the original experience. We set up our experiments by aiming to investigate the post-emotional processing of experiences that resemble daily life occurrences. This premise also guided our previous research work (Curci et al., 2013), of which the present paper represents a continuation. As we reviewed in the preceding pages, a large body of evidence concerning intrusions and rumination has been provided by studies on traumas. However, the emotional manipulation adopted in the present experiments raises the problem of the generalizability of our findings to real life contexts. Comparing the outcomes of processing less vs. more extreme emotional daily life experiences should be the focus of future investigation.

To conclude, our findings can be summarized as follows: exposure to verbal negative material impairs verbal performance, while exposure to visual negative material results in an impairment of both verbal and visuospatial performance. Intrusions were only affected by the emotional valence of the original experience, while performing a visuospatial task resulted in enhanced rumination only after exposure to verbal material. These findings account for the idea that emotional processing requires a balance of cognitive resources spreading over time and influenced by the interaction between the modality of cognitive tasks in which the individual is engaged and the nature (verbal vs. visual) of the triggering emotional experience. Furthermore, the current results confirm that, although they are very similar phenomena, sensory intrusions and rumination differ somewhat in their determinants and evolution. We suggest that explicative modeling of emotional elaboration needs to be integrated with a consideration of this evidence.

### Conflict of Interest Statement

The authors declare that the research was conducted in the absence of any commercial or financial relationships that could be construed as a potential conflict of interest.

## Footnotes

1 Unlike our previous study on the same subject (Curci et al., 2013), in designing the present experiments we adopted the ERRI indices in order to have a validated measure of the different facets of the construct of rumination. Although, as accounted by the authors of the scale (Cann et al., 2011), some overlaps exists between the ERRI and IES-R dimensions, the ERRI scales are intended to capture some unique variance corresponding to the presence of some elaborative thoughts (either intrusive or deliberate), without implying the persistence of distress symptoms, as the IES-R does.

2 The IES-R (Weiss and Marmar, 1996) also includes a third scale of symptoms of emotional distress, Hyperarousal, which was not relevant for the purposes of the present studies, and thus was not considered in our analyses.

3 To consider the role of individual dispositions on the development of intrusions and ruminations, the analyses were repeated by introducing the RRS indices as covariates. A significant association was found only for the scale of Brooding [*F*s (1, 115) > 11.00, *p* ≤ 0.001, partial η^2^ > 0.09]. However, the pattern of significance for the effects of Emotion valence, Task modality, and Follow-up did not change after introducing such a covariate.

4 In order to assess the role of individual dispositions on the persistence of intrusions and rumination, the analyses were repeated by introducing the RRS indices as covariates. A significant association was found only for the scale of Brooding on the ERRI indices [*F*s (1, 158) > 13.00, *p* ≤ 0.001, partial η^2^ > 0.08], but the pattern of significance for the effects of Emotion valence, Task modality, and Follow-up did not change after introducing such a covariate.
